# Resistance to high-fat diet-induced weight gain in transgenic mice overexpressing human wild-type α-synuclein: A model for metabolic dysfunction in Parkinson’s disease

**DOI:** 10.21203/rs.3.rs-4870881/v1

**Published:** 2024-08-30

**Authors:** K.C. Biju, Enrique Torres Hernandez, Alison Michelle Stallings, Ada C. Felix-Ortiz, Skanda K. Hebbale, Luke Norton, Michael J. Mader, Robert A. Clark

**Affiliations:** a South Texas Veterans Health Care System, 7400 Merton Minter Blvd, San Antonio, Texas 78229; b Department of Medicine, The University of Texas Health Science Center at San Antonio, 7703 Floyd Curl Drive, San Antonio, Texas 78229

## Abstract

Unintentional weight loss, primarily due to the loss of fat mass rather than muscle mass, is common among patients with Parkinson’s disease (PD) and is associated with poor quality of life and accelerated disease progression. Since transgenic mice overexpressing human wild-type α-synuclein (α-Syn mice) are modestly leaner than control mice, and since diabetes, a metabolic disorder, is a major risk factor for PD, we reasoned that high-fat diet-induced diabetes/metabolic dysregulation in α-Syn mice may serve as a robust tool for exploring how early α-synuclein pathology contributes to metabolic dysregulation, leading to weight loss in PD. Thus, α-Syn and age-matched controls were fed a high-fat diet (HFD) chow (60% fat calories) *ad libitum* for four months. Compared with controls on HFD (control-HFD), α-Syn mice on HFD (α-Syn-HFD) were dramatically leaner. The resistance to gaining weight in α-Syn-HFD mice was accompanied by improved glucose tolerance, a dramatic decrease in fat mass, and an increase in energy expenditure. Despite this leaner phenotype and better glucose tolerance, the mortality was much higher in male α-Syn-HFD mice than in all controls, but was unaffected in females, suggesting protective effects of female sex hormones, as well as lower α-synuclein levels. Immunoblot analysis of insulin signaling in the olfactory bulb, the proposed initial seeding site of α-synuclein pathology, revealed a decrease of IGF-IRβ, p GSK, and p mTOR in α-Syn-HFD mice. Since GSK-3β and mTOR regulate synaptic plasticity, we assessed levels of PSD-95 and synaptophysin in the olfactory bulb. As anticipated, we observed a significant decrease in the levels of PSD-95, along with a potentially compensatory increase in synaptophysin levels. Our results show that α-Syn mice, when challenged with diet-induced diabetes/metabolic dysregulation, clearly reveal a profile of robust metabolic dysfunction, thus providing a sensitive tool for assessing the underlying mechanism of metabolic dysfunction and its impact on weight loss and disease progression in PD. We propose a role for olfactory dysfunction in PD-related unintentional weight loss and suggest that strategies aimed at increasing body weight/BMI will improve the quality of life and prognosis for people living with PD.

## INTRODUCTION

Although Parkinson’s disease (PD) is generally associated with its characteristic movement dysfunction, non-motor symptoms add substantially to the overall level of disability and can seriously impact quality of life^1^. For example, unintentional weight loss, primarily due to the loss of fat mass rather than muscle mass^2^, is common among patients with PD^3–8^. Risks associated with this non-motor feature include malnutrition, frailty, falls, fractures, and death. Many PD patients with unintentional weight loss are particularly troubled by the combination of bradykinesia, postural instability, tremor, and urinary urgency, which increases their chances of falls and fractures, thus significantly impacting quality of life, risk of early institutionalization, and health economic status. Unintentional weight loss often predates PD motor symptoms by several years^3,9^, perhaps comprising an unrecognized manifestation of prodromal PD. Weight loss in PD patients does not appear to be caused by reduced energy intake. In fact, in a cross-sectional study, despite equivalent dietary intake, PD patients had lower whole-body fat mass and percentage compared with controls, even after accounting for the effects of age and gender^6^. Longitudinal data from 1673 participants in the National Institute of Neurological Disorders and Stroke (NINDS) Exploratory Trials in Parkinson’s Disease Long-term Study 1 showed that patients with a decreasing body mass index (BMI) had higher motor and total Unified Parkinson’s Disease Rating Scale (UPDRS) scores than the patients with a stable BMI (despite optimal treatment), whereas those with an increasing BMI had lower motor and total UPDRS scores than patients with a stable BMI^10^. This indicates that strategies aimed at increasing body weight/BMI would likely improve the quality of life and prognosis for people living with PD. However, the pathophysiological mechanisms underlying weight loss and its relationship to other PD symptoms are poorly understood. Importantly, despite the impact of weight loss on worsening UPDRS scores, the nature of the link between weight loss and dopaminergic neurodegeneration itself remains unknown. Finally, we do not know how the α-synuclein accumulation that is central to PD pathogenesis impacts metabolism and energy homeostasis, ultimately leading to weight loss.

Male transgenic mice (Line 61) overexpressing human wild-type α-synuclein have been reported to be leaner than littermate control male mice^11^, indicating that α-synuclein has a causal role in weight loss in PD. Since dysregulation of glucose metabolism is a very prominent early manifestation of PD^12,13^ and diabetes, a disorder of glucose metabolism, is a major risk factor for PD^14^, we reasoned that high-fat diet-induced diabetes/obesity in transgenic mice overexpressing human wild-type α-synuclein (henceforth, α-Syn mice) may serve as a tool for discovering how early α-synuclein pathology contributes to metabolic dysregulation, leading to weight loss in PD. Thus, both male and female α-Syn and age-matched control mice were fed a high-fat diet chow (60% fat calories) *ad libitum*. Our results show that α-Syn mice, when challenged with diet-induced obesity, reveal a clear profile of robust metabolic dysfunction, thus providing a sensitive tool for assessing the underlying mechanism of metabolic dysfunction and its impact on weight loss and disease progression in PD. We also provide a theoretical explanation for weight loss in PD by linking metabolism and energy homeostasis with olfactory dysfunction in PD, based on the following: (1) the olfactory system is a metabolic sensor of brain insulin and glucose levels, playing a role in controlling energy homeostasis in response to both sensory (external) and hormonal (internal) signals^15,16^; (2) α-synuclein pathology in PD likely first appears in the olfactory bulb^17,18^; and (3) impaired sense of smell is very often the first manifestation of prodromal PD^19^.

## RESULTS

### α-Syn mice are leaner compared with littermate controls

**Fig. 1:**
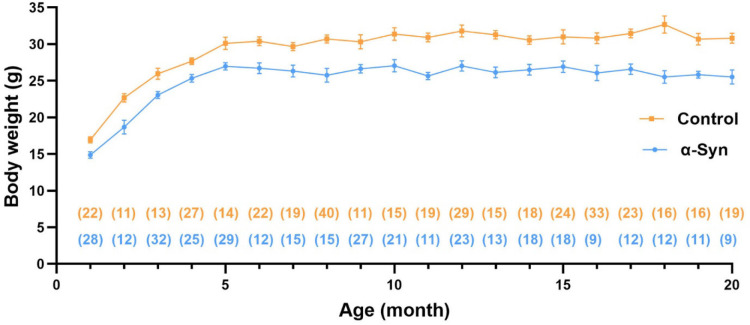
The α-Syn mice exhibit a leaner phenotype across all age groups studied. Data represent mean ± SEM. The number of mice for each group is shown in parentheses. Since the data were collected cross-sectionally and randomly over a period of seven years, statistical analysis was performed separately for each time point using unpaired t-tests. *P* < 0.005 for all age groups.

Starting at 1 month of age (post-weaning), the body weights of α-Syn mice and controls were recorded monthly until 20 months of age. The body weights were measured cross-sectionally and randomly across multiple generations over a period of seven years. Only male mice were used for this initial body weight analysis. The α-Syn mice were significantly (*P* < 0.005) leaner compared with littermate controls across all age groups (Fig. 1). At 1 month of age, the α-Syn mice weighed, on average, 9.4% less than the age-matched controls. Both strains of mice gained weight at a somewhat rapid pace until 5 months of age, with α-Syn mice maintaining the initial percentage of weight difference. At 5 months of age body weight was somewhat stabilized in both strains, but, over time the difference in their body weight slowly increased, with 20-month-old α-Syn mice being, on average, 17.1% lighter than controls.

### α-Syn mice are resistant to high-fat diet-induced weight gain

**Fig. 2:**
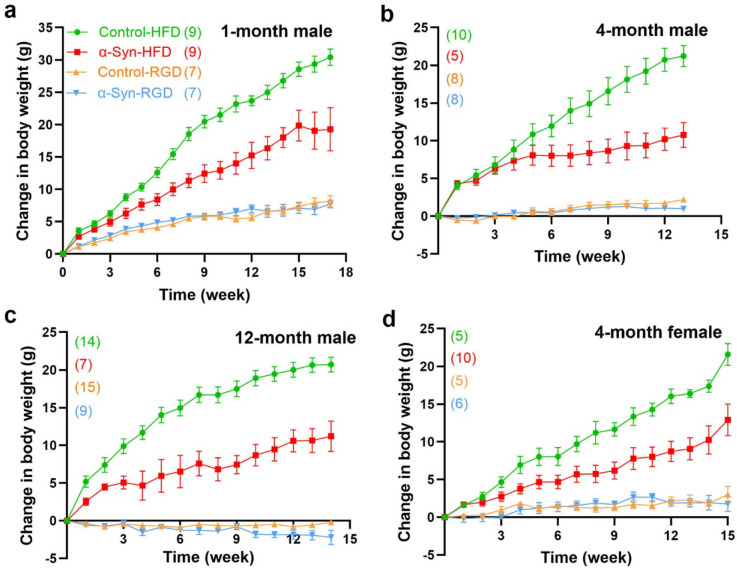
The α-Syn mice on a high-fat diet exhibit resistance to gaining body weight. Mean change from initial body weights of 1-month male (**a**), 4-month male (**b**), 12-month male (**c**), and 4-month female (**d**) mice, expressed ± standard error. The number of mice for each group is shown in parentheses. Statistical analysis was performed separately for the high-fat diet (*P* < 0.0001 for **a**-**d**) and regular diet groups, using two-way repeated measures ANOVA. For the regular diet groups, the weight difference did not reach statistical significance for the 1-month male (*P* = 0.2127) and 4-month female (*P* = 0.3355) groups, whereas the differences were statistically significant for the 4- and 12-month male groups (*P* < 0.0001).

**Fig. 3:**
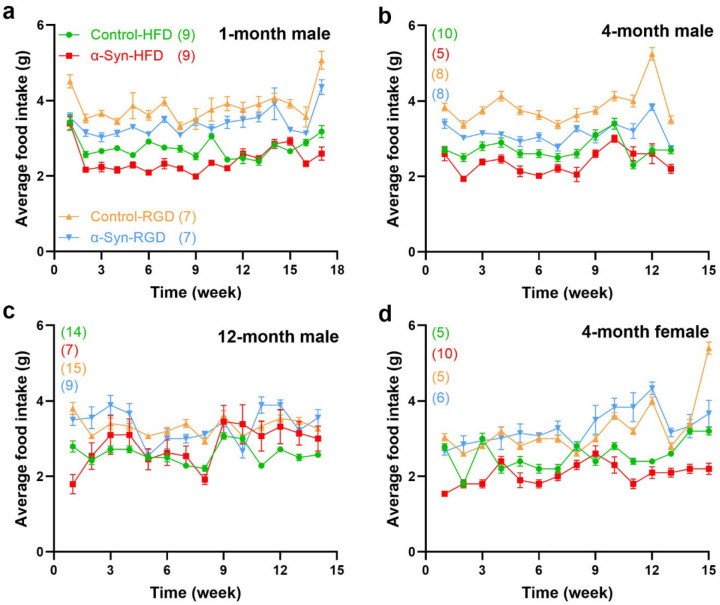
Analysis of food intake. Average daily food intake was measured weekly. Although the differences in the levels of food intake between α-Syn and control mice in the high-fat and regular diet groups were only modest, they were significant (*P* < 0.0001), except for that of the 1-month male regular diet sub-group (*P* = 0.3351). The number of mice for each group is shown in parentheses. Statistical analysis was performed separately for the high-fat diet and regular diet groups using two-way repeated measures ANOVA.

**Fig. 4:**
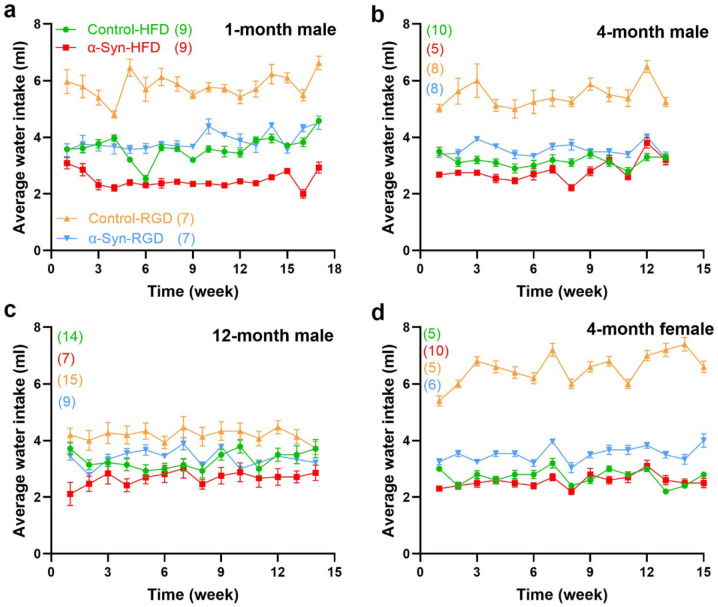
Analysis of water intake. Average daily water intake was measured weekly. The number of mice for each group is shown in parentheses. Statistical analysis was performed separately for the high-fat diet and regular diet groups in **a**-**d**, using two-way repeated measures ANOVA. *P* < 0.009 for all groups.

We hypothesized that high-fat diet (HFD)-induced type 2 diabetes (a major PD risk factor) may be informative regarding the linkages between α-synucleinopathy and metabolic dysfunction/weight loss in PD. Thus, three separate groups of male α-Syn mice and age-matched controls were fed a high-fat chow diet (60% fat calories) *ad libitum* starting at 1, 4, and 12 months of age to assess the impact of aging and/or disease severity. Similarly, another three separate groups of male α-Syn mice and age-matched controls were fed a regular chow diet (RD). To assess sex-specific effects, a group of female mice was similarly fed a high-fat chow diet starting at 4 months of age. Compared with controls on HFD (control-HFD), α-Syn mice on HFD (α-Syn-HFD) were dramatically leaner (Fig. 2), despite somewhat similar levels of food (Fig. 3) and water intake (Fig. 4) in the HFD groups. Note that some of the subjects died (see Fig. 6) at various time points before the analysis time window ended and were excluded. The weight difference between α-Syn-HFD and control-HFD groups was apparent within 1 week post-HFD in the 12-month male group, whereas in the remaining groups the difference was evident within 3–4 weeks post-HFD (Fig. 2). Although the 1-month male and 4-month female α-Syn mice on regular chow diet in general weighed less than controls on the same diet, unlike the cross-sectional body weight analysis represented in Fig. 1, the differences were not statistically significant (*P* = 0.2127 for 1-month male and *P* = 0.3355 for 4-month female), possibly due to the amount of variation inherent in measurement of bodyweight for this small sample size. Similarly, the water intake for the younger (1- and 4-month) control mice on regular chow diet was much higher (Fig. 4a, b and d), compared with other groups; however, body weights are unlikely to be influenced by the higher water intake in these younger groups since the correlation between fluid consumption and body weight is small^20^.

### α-Syn mice are resistant to high-fat diet-induced impaired glucose tolerance/insulin resistance

**Fig. 5:**
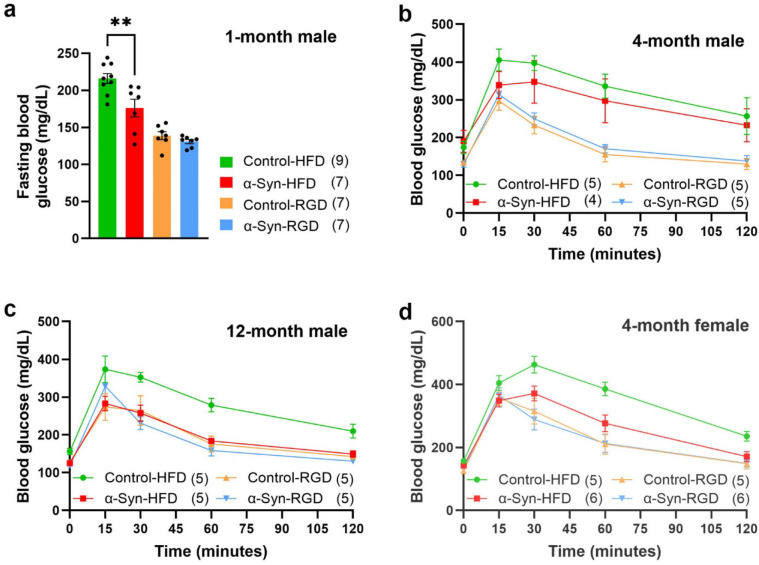
α-Syn mice on high-fat diet exhibit faster clearance of blood glucose. Fasting blood glucose levels in 1-month male group (**a**) and IPGTT in 4-month male (**b**), 12-month male (**c**) and 4-month female (**d**) groups after 14–16 weeks of high-fat diet treatment. The number of mice for each group is shown in parentheses. Statistical analysis was performed separately for the high-fat diet (*P* = 0.0085) and regular diet sub-groups (*P* = 0.2181) in the 1-month male group using unpaired t-tests. For the 4-month male and female groups, analysis was performed using the mixed effects model (*P* < 0.0001) due to unequal sample sizes. Three-way repeated measures ANOVA was used for the 12-month male group due to equal sample sizes (*P* < 0.0001).

In line with the body weight data, α-Syn mice on HFD exhibited lower fasting blood glucose levels and a more rapid clearance of blood glucose during the intraperitoneal glucose tolerance test (IPGTT) than control-HFD mice (Fig. 5). There was also a striking influence of age (disease progression) and sex on the temporal dynamics of glucose clearance from the blood. Although the 4-month male α-Syn-HFD mice had better glucose tolerance than age-matched control-HFD mice, their glucose levels did not return to baseline at 120 minutes (Fig. 5b); however, the glucose levels of the 4-month α-Syn-HFD female mice did return nearly to baseline by this time (Fig. 5d). Unlike the 4-month male α-Syn-HFD mice, the glucose tolerance of 12-month male α-Syn-HFD mice (Fig. 5c) was indistinguishable from that of control mice on a regular diet. Note that in the 1-month males, we measured only fasting glucose levels, which indeed were lower in α-Syn-HFD mice than in control-HFD mice (Fig. 5a).

### High-fat diet-induced increased mortality in α-Synuclein mice

**Fig. 6:**
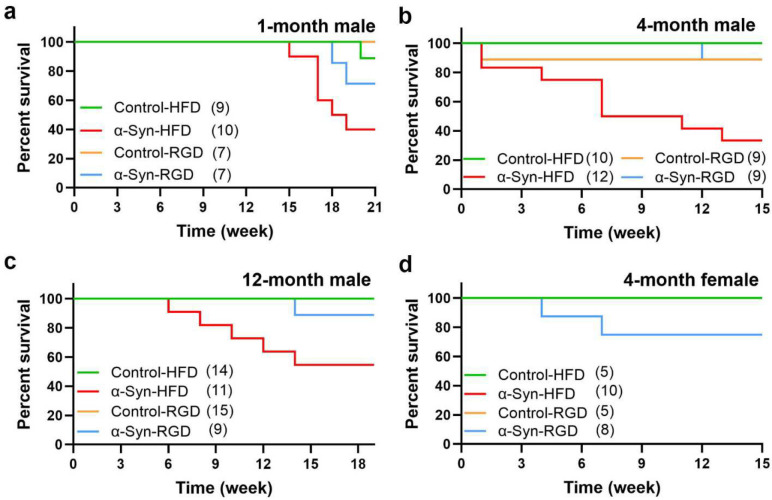
Analysis of mortality. Kaplan-Meier survival curves, showing increased mortality in male α-Syn mice on high-fat diet. The number of mice for each group is shown in parentheses. Statistical analysis was performed using the Log-rank (Mantel-Cox) test. *P* = 0.0108 (**a**), *P* < 0.0005 (**b** and **c**), and *P* = 0.1482 (**d**).

**Fig. 7:**
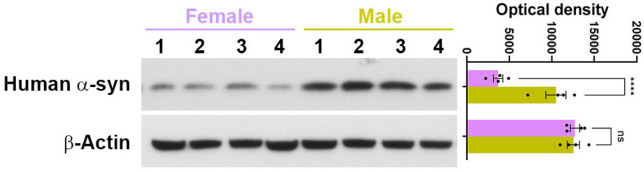
Female α-Syn mice have lower brain human α-synuclein protein levels, compared with males. Western blot analysis of α-synuclein levels in the olfactory bulbs of 4-month female and male α-Syn mice, using antibodies specific to human α-synuclein. Statistical analysis was performed using unpaired t-test (two-tail) with Welch correction. ****, *P* < 0.0001. ns, nonsignificant.

Despite the leaner phenotype and better glucose tolerance, the death rate in male α-Syn mice on HFD was much higher in all age groups, albeit less stark in 1-month-old males; in the 1-month-old male group, which exhibited increased mortality only after 15 weeks into HFD treatment (Fig. 6). By the end of HFD treatment (15 – 21 weeks), about 55 – 60 percent of the male α-Syn mice had died in all age groups. However, unlike the males, female α-Syn-HFD mice did not exhibit increased mortality (Fig. 6d), likely due to reduced human α-synuclein protein levels in the brains of female α-Syn mice (Fig. 7)

### High-fat diet-induced altered body composition in α-Syn mice

**Fig. 8:**
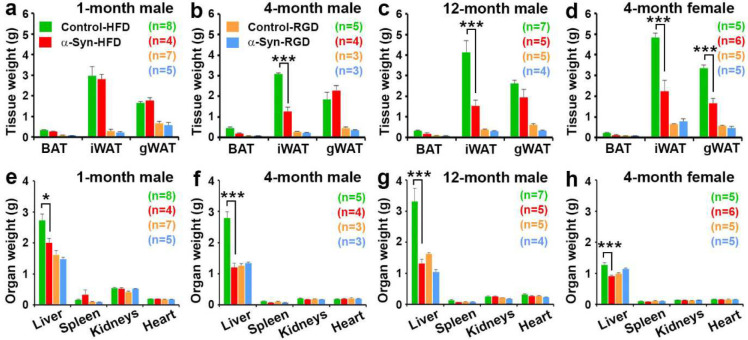
Body composition measured by organ weight. Fat composition was measured by weighing brown adipose tissue (BAT), subcutaneous inguinal white adipose tissue (iWAT), and gonadal white adipose tissue (gWAT). The weights of organs other than liver were unaffected. The number of mice for each group is shown in parentheses. Statistical analysis for each type of fat mass and organ was performed separately for the high-fat diet and regular diet sub-groups using unpaired t-tests. *, *P* < 0.0431 and ***, *P* < 0.0001.

**Fig. 9:**
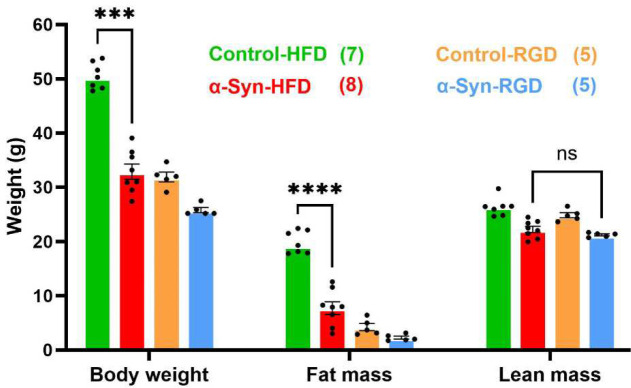
Body composition measured by qMRI. The high-fat diet did not affect significantly (*P* = 0.3277, Control-HFD vs. Control-RGD and *P* = 0.6876, α-Syn-HFD vs. α-Syn-RGD) the lean mass of either genotype. The number of mice for each group is shown in parentheses. Statistical analysis was performed using unpaired t-tests, comparing α-Syn and Control mice separately for each diet group. ***, *P* = 0.0002; ****, *P* < 0.0001.

The slow weight gain of α-Syn-HFD mice reflected a significantly reduced fat mass, compared with control-HFD mice. In particular, the subcutaneous inguinal fat (iWAT) showed the biggest difference between the two groups (Fig. 8a–d). Since the food intake was similar among HFD groups, the results indicate that α-Syn mice have enhanced fat-burning capacity. In addition, since the food intake was similar and the body weight was only modestly, albeit significantly (*P* < 0.0001; Fig. 1), different between the α-Syn and control mice on regular diet, it is unlikely that the α-Syn mice had a deficit in nutrient absorption. In addition, there was a near doubling of liver weight in 4- and 12-month-old male control HFD mice but none in α-Syn-HFD mice (Fig 8f and g). The influence of HFD on liver weight was gender-selective in that only a modest increase was observed in female control-HFD mice (Fig 8h). Since most of the mice with lower body weight died before we were able to assess fat mass, the difference in fat mass between the two 1-month male groups on HFD was not large and their liver weight showed only a modest contrast. Using a new cohort of 4-month males, we assessed body composition using quantitative MRI (qMRI); the data confirm that the resistance to weight gain in α-Syn-HFD mice was due to reduced fat mass rather than reduced lean mass (Fig. 9). The HFD did not affect significantly (*P* = 0.3277, Control-HFD vs. Control-RGD and *P* = 0.6876, α-Syn-HFD vs. α-Syn-RGD) the lean mass of either strain of mice.

### Evidence of altered insulin signaling in α-Syn mice on high-fat diet

**Fig. 10:**
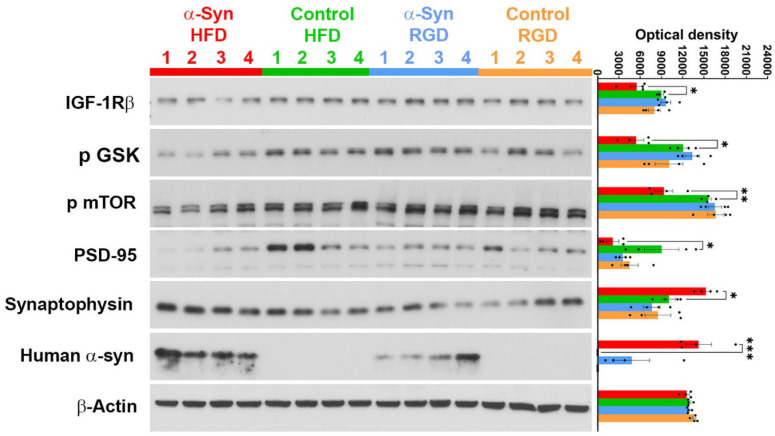
Molecules involved in insulin signaling are altered in the olfactory bulb of α-Syn-HFD mice. Western blot analysis of regulators of insulin signaling and synaptic plasticity in the olfactory bulb of the 4-month male group. Statistical analysis was performed using unpaired t-tests, comparing α-Syn and Control mice separately for each diet group. *, *P* = 0.0095 (IGF-IRβ), *P* = 0.0053 (p GSK), *P* = 0.0006 (p mTOR), *P* = 0.0293 (PSD-95), *P* = 0.0340 (synaptophysin), and *P* = 0.0001 (human α-synuclein).

Since the olfactory system is a metabolic sensor of brain insulin and glucose levels, participating actively in the modulation of peripheral metabolism in response to both external and internal cues^15,16^, we assessed, in the 4-month male group, insulin signaling in the olfactory bulb using the Insulin/IGF-1 Signaling Pathway Antibody Kit (Cell Signaling Cat# 42022). Of the nine selected insulin signaling molecules assessed, levels of Type I insulin-like growth factor receptor β (IGF-IRβ), glycogen synthase kinase-3β (GSK-3β) phosphorylated at serine 9, and mechanistic target of rapamycin (mTOR) phosphorylated at serine 2448 were significantly (*P* = 0.0095, *P* = 0.0053, and *P* = 0.0006 for IGF-IRβ, p GSK, and p mTOR, respectively) lower in α-Syn-HFD mice (Fig. 10). It has been shown that induction of insulin signaling with IGF-1 suppresses α-synuclein aggregation and toxicity^21^. Accordingly, the decrease in IGF-IRβ was accompanied by an increase in α-synuclein levels in α-Syn-HFD mice (Fig. 10). GSK-3β and mTOR regulate synaptic plasticity by controlling synapse density and regulation of postsynaptic density protein 95 (PSD-95)^21–23^. As predicted, we observed a significant (*P* = 0.0293) decrease in the levels of PSD-95 in the olfactory bulb of α-Syn-HFD mice (Fig. 10). A decrease in PSD-95 can lead to an increase in presynaptic protein synaptophysin levels, considered to be a compensatory mechanism in deficient synaptic transmission^24^, which is proposed to be an early event in PD-related neurodegeneration. Therefore, we assessed synaptophysin levels in the olfactory bulb, the proposed induction/initial seeding site of α-synuclein pathology, and as expected the levels increased in α-Syn-HFD mice (Fig. 10).

### Substrate utilization and energy expenditure in α-Syn mice

**Fig. 11:**
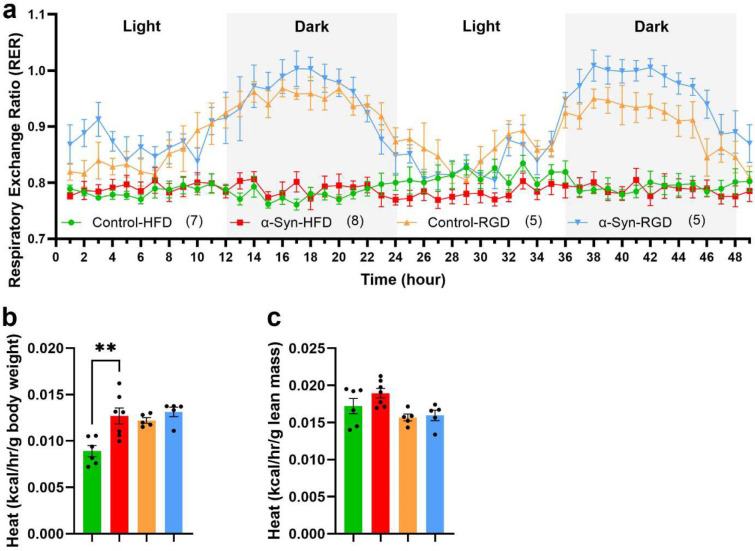
Resistance to weight gain in α-Syn-HFD mice is accompanied by an increase in energy expenditure. Indirect calorimetry data showing 48-hour respiratory exchange ratio (**a**). Energy expenditure, represented as heat, is normalized to both total body weight (**b**) and lean mass (**c**). The number of mice for each group is shown in parentheses. Statistical analysis was performed using a mixed effects model (*P* < 0.0001), due to unequal sample sizes, for respiratory exchange ratio (**a**), and using independent unpaired t-tests, comparing α-Syn and Control mice separately for each diet group, for energy expenditure (**b** and **c**). **, *P* = 0.0023

The 4-month male group was subjected to 48-hour indirect calorimetry to calculate respiratory exchange ratio (RER) and energy expenditure. An RER of about 0.7 indicates that fats are being used as the main fuel for the body, whereas an RER of 1 indicates that carbohydrates are being used. An RER between 0.7 and 1 indicates a mix of fat and carbohydrates. The energy substrates of HFD groups were predominantly fat during both the light and dark (active) cycles, whereas RGD groups switched from predominantly fat during the light cycle to predominantly carbohydrates during the dark cycle (Fig. 11a). Interestingly, during the dark cycle, α-Syn-RGD mice tended to utilize substantially more carbohydrates than Control-RGD mice. There was an effect of body weight on energy expenditure; consistent with resistance to weight gain, the energy expenditure in α-Syn-HFD mice was significantly higher (*P* = 0.0023), compared with Control-HFD mice, when normalized to total body weight (Fig. 11b). Although a similar trend was observed when energy expenditure was normalized to lean mass (Fig. 11c), the difference was not significant (*P* = 0.3471), possibly because it was insufficiently powered statistically. However, the energy expenditure, normalized to lean mass, in α-Syn-HFD was significantly higher (*P* = 0.0301) than that of the regular chow diet group, indicating that the increased energy expenditure contributed to resistance to weight gain in α-Syn-HFD mice.

## DISCUSSION

We demonstrate here that α-Syn transgenic mice, when challenged with high-fat diet, clearly reveal a profile of robust metabolic dysfunction, indicating a relationship between abnormal α-synuclein accumulation and the dysregulation of energy homeostasis in this well-studied model of Parkinson’s disease (PD). α-Syn mice fed with high-fat diet could thus serve as models for understanding the metabolic dysfunction and associated weight loss that significantly impact the quality of life of PD patients.

Unintentional weight loss, primarily due to the loss of fat mass rather than muscle mass^2^, is common among patients with PD^3–5,7,8^, often predating the development of clinical PD by several years^3^. In line with these findings, dysregulation of glucose metabolism has been found to be a very prominent early manifestation of PD^12,13^. Despite their heavy toll on patients and caregivers, metabolic dysfunction, unintentional weight loss, and disproportionate fat loss in PD are not well understood, and their cause-and-effect relationship with α-synuclein pathology is unclear.

Based on available literature and the data presented here, we propose a hypothesis linking metabolic dysfunction and unintentional weight loss with olfactory dysfunction in PD. Sense of smell is often the first casualty of PD, predating the development of clinical PD by as much as 20 years^19^. Over 90% of PD patients exhibit olfactory dysfunction^25^ and there is strong evidence that α-synucleinopathy, one of the hallmarks of PD, first appears in the olfactory system^17,18^. Recent evidence shows that, in addition to sensing external chemical cues, the olfactory system is a metabolic sensor of brain insulin and glucose levels, playing a key role in controlling energy homeostasis in response to both sensory (external) and hormonal (internal) signals^15,16^. The exact mechanisms by which olfaction influences metabolism are unknown. Riera and colleagues^26^ recently demonstrated that upon inducing hyposmia (reduced olfaction) in mice, the animals become resistant to diet-induced obesity, and conversely, that enhancing their sense of smell leads to increased adiposity. The researchers further showed that the leaner phenotype associated with hyposmia was accompanied by increased lipolysis via upregulation of uncoupling protein 1 (UCP1)^26^. Consistent with this report, a recent study^27^ showed that female olfactory cues reduced body weight in males by enhancing fat burning via UCP1 upregulation. Lastly, in a human study, the use of a novel self-administered nasal device to reduce olfactory sensitivity (induced hyposmia) resulted in significant weight loss^28^. Whether it is food odor (main olfactory system^26^) or social odor (vomeronasal^27^), these reports corroborate the fact that olfaction plays a role in modulating body weight by regulating lipolysis.

A reciprocal communication exists between olfactory sensitivity and energy homeostasis, as sensitivity increases in a fasted state and decreases in a sated state^16,26^. This suggests that hyposmia/loss of smell (as seen in PD) could skew the brain’s homeostatic response towards a “satiety state-like” metabolic response, thus leading to catabolic energy utilization/lipolysis. This could partly explain unintentional weight loss in PD, as well as resistance to diet-induced weight gain in α-Syn mice, which also exhibit olfactory dyfunction and olfactory bulb α-synuclein pathology similar to that seen in PD patients^29^. Indeed, a significant correlation was found between olfaction and BMI in human PD patients, indicating that hyposmia, but not hypogeusia, contributes to weight loss in PD^30^. In further support of a role for olfaction in PD-related metabolic dysfunction and weight loss, we provide evidence of altered insulin signaling in α-Syn-HSD mice. Levels of IGF-IRβ, GSK-3β phosphorylated at serine 9 and mTOR phosphorylated at serine 2448 were significantly lower in α-Syn-HFD mice. Both GSK-3β and mTOR regulate several elements downstream of IGF-IRβ that are typically disrupted in PD, including apoptosis, autophagy, neuronal metabolism, protein synthesis, and synaptic plasticity^21^. Indeed, GSK-3β and mTOR signaling are altered in PD^31,32^. GSK-3β and mTOR regulate synaptic plasticity by controlling synapse density and regulation of postsynaptic density protein 95 (PSD-95)^21–23^. As anticipated, there was a significant decrease in the levels of PSD-95 in the olfactory bulbs of α-Syn-HFD mice, and this was accompanied by an increase in synaptophysin levels. A decrease in PSD-95 can lead to an increase in presynaptic protein synaptophysin levels, considered a compensatory mechanism in deficient synaptic transmission^24^, which is proposed to be an early event in PD-related neurodegeneration. Taken together, these data indicate that GSK-3β and mTOR are likely two common denominators that link metabolic dysfunction with weight loss and neurodegeneration, and thus offer a molecular basis for the association between weight loss and disease severity seen in PD patients^10^.

Several of our observations support the notion that resistance to high-fat diet-induced weight gain in α-Syn mice is due to enhanced fat-burning capacity and energy expenditure:

1. Body composition analysis using qMRI confirmed that the resistance to weight gain in α-Syn-HFD mice was due to reduced fat mass rather than reduced lean mass. Interestingly, weight loss in PD occurs primarily due to fat rather than muscle loss^6^.
2. Fat composition analysis by weighing BAT, iWAT, and gWAT at the end of the study not only confirmed the qMRI data but further revealed that the biggest reduction occurred for iWAT in 4- and 12-month-old male α-Syn-HFD mice, whereas both iWAT and gWAT were significantly reduced in female α-Syn-HFD mice, compared with control-HFD mice.
3. The resistance to weight gain and reduced fat mass in α-Syn-HFD mice reflected a more rapid clearance of blood glucose during the intraperitoneal glucose tolerance test. Similar to the composition of fat mass, there was an effect of sex on the temporal dynamics of blood glucose clearance during the intraperitoneal glucose tolerance test. Although the 4-month-old male α-Syn-HFD mice had better glucose tolerance than age-matched control-HFD mice, the glucose levels of the male α-Syn-HFD mice did not return to baseline or near baseline at 120 minutes; however, the glucose levels of the equivalent female mice did return to near baseline at this time. Although the exact reason for this sex difference is unclear, it probably reflects the difference in the composition of fat mass in male vs. female α-Syn-HFD mice. Most men accumulate excess calories in visceral adipose tissue (VAT), whereas women in general equivalently expand VAT and subcutaneous adipose tissue (SAT) in response to over-nutrition^33,34^. SAT produces more leptin and is less inflamed and less insulin-resistant than VAT, likely explaining why generally women are more insulin sensitive. Interestingly, along with the improved glucose tolerance, subcutaneous iWAT was the largest in female α-Syn-HFD mice, as opposed to visceral gonadal WAT (gWAT) in age-matched males.
4. High-fat diet typically leads to a dramatic increase in fat accumulation in hepatocytes; accordingly, there was a near doubling of liver weight in 4- and 12-month-old male control-HFD mice, and this increase in liver weight was absent in α-Syn-HFD mice; a similar effect was also seen in the 1-month-old male and 4-month-old female mice, albeit to a lesser degree. Hepatocytes accumulate fat when the cellular input of fatty acids via either ingestion (here high-fat diet) or hepatic lipogenesis surpasses fatty acid output via oxidation or export^35^. Although the α-Syn-HFD mice were protected against the increase in liver weight, the α-Syn-HFD groups had a relatively higher fat mass, compared with regular diet chow groups. This, along with the fact that ingestion of high-fat diet chow was similar in control and α-Syn mice, indicates that a combination of fatty acid oxidation and export prevented hepatic fat accumulation and thus the increase in liver weight in α-Syn-HFD mice.
5. Lastly, calorimetry data revealed that energy expenditure normalized to body weight was significantly higher in α-Syn-HFD mice than in control-HFD mice. However, the increase in energy expenditure in α-Syn-HFD mice did not reach statistical significance when normalized to lean mass. It is noteworthy that energy expenditure is influenced by physical activity levels. Since our Oxymax system was not equipped for concomitant measurement of activity levels, the effect of activity on energy expenditure could not be determined. Despite this limitation, the fact that energy expenditure normalized to lean mass in α-Syn-HFD was significantly higher than that of the regular chow diet group indicates that the increased energy expenditure contributed to resistance to weight gain in α-Syn-HFD mice.

Although the α-Syn-HFD mice exhibited a leaner phenotype and better glucose tolerance, the death rate in male α-Syn-HFD was much higher in all age groups. The exact reason for this increased mortality is unclear; however, it is possible that the rapid clearance of glucose from blood during the IPGTT may reflect abnormal glucose metabolism and a cellular energetics deficit that aggravates oxidative stress, neuroinflammation, and neurodegeneration, ultimately leading to death. If this hypothesis is correct, one would also expect increased mortality in female α-Syn-HFD mice, along with leaner phenotype and faster clearance of glucose from the blood; however, unlike the males, female α-Syn-HFD mice did not exhibit increased mortality. This effect could result from the combination of female sex hormones and lower brain α-synuclein levels.

Male α-Syn (Line 61) mice on regular chow diet have previously been reported to be relatively leaner with no significant change in glucose or insulin response in IPGTT, compared with wild-type male controls^11^, suggesting a modest metabolic alteration in α-Syn mice. The data from the current study not only corroborate the previous report^11^ but further show that high-fat diet is an effective way to enhance mild metabolic phenotypes in α-Syn mice in order to elucidate the relationship between pathological α-synuclein aggregation and the dysregulation of energy homeostasis that leads to weight loss and accelerated disease progression in PD. To this end, we provide a novel hypothesis linking metabolic dysfunction in PD with olfactory dysfunction.

## METHODS

### Mice

Transgenic mice overexpressing human wild-type α-synuclein under the direction of a mouse *Thy-1* promoter (mThy1-hSNCA; Line 61)^36^ in C57BL/6J background and non-transgenic littermate controls, both male and female, were used. The mice were housed in standard plexiglass cages with 7099 TEK-Fresh animal bedding (Envigo, Indianapolis, IN) and were fed either a high-fat diet chow (60% fat calories; Cat # D12492; Research Diets, New Brunswick, NJ) or a standard Teklad irradiated LM-485 mouse diet (Cat # 7912; Envigo, Indianapolis, IN) *ad libitum* and with full-time access to acidified drinking water. The mice were maintained in a 12/12 hour light/dark cycle at 24°C room temperature and 50–55% humidity. Animal husbandry was in accordance with the National Institutes of Health Guide for the Care and Use of Laboratory Animals and the Society for Neuroscience Policies on the Use of Animals and Humans in Research. All experimental procedures were approved by the Institutional Animal Care and Use Committee at the South Texas Veterans Health Care System, Audie L. Murphy Division, and the University of Texas Health Science Center, San Antonio. All live animal testing was performed during the light cycle with 30–40 lux light intensity maintained throughout the tests. The smallest possible number of mice was used (based on power calculations) and all efforts were made to minimize suffering. All experiments were randomized and performed blind-coded whenever feasible, although for all live animal testing weight gain made it obvious to the experimenter which animals was fed with high-fat diet chow.

### Body weight

Body weight was measured between 10 AM and noon once a week. For the purpose of clarity, cumulative changes in body weight are represented in Fig. 2.

### Food and water intake

Twenty four-hour food intake was measured manually as the difference between the pelleted chow put into the cage and that remaining at the end of 24 hours. Measurements were taken between 5 PM and 6 PM, and the chow crumbs that had fallen into the bedding were accounted for. Water intake was measured by weighing the water bottle.

### Intraperitoneal glucose tolerance test

Mice were fasted for 5 hours and then their tails were bled to measure fasting glucose concentrations using a glucometer (Contour next EZ). Immediately after measuring fasting glucose concentrations, the mice were injected, intraperitoneally, with 500 μl of 10% Dextrose USP (B. Braun Medical Inc., Bethlehem, PA) and blood glucose concentrations were determined 15, 30, 60 and 120 minutes later.

### Indirect calorimetry

Indirect calorimetry studies were performed using an eight-chamber Oxymax-Comprehensive Lab Animal Monitoring System (CLAMS; Columbus Instruments, Columbus, OH) at the Integrative Physiology and Aging Core of the San Antonio Nathan Shock Center, UTHSCSA. The mice were acclimated to the system for 24 hours prior to collecting various measurements of indirect calorimetry for 48 hours. Food and water were provided *ad libitum*.

### Body composition

Body composition was assessed by quantitative MRI (EchoMRI LLC, Houston, TX), and total fat mass, lean mass, and free water were calculated. At the end of the study the mice were euthanized and BAT, iWAT and gWAT, liver, spleen, heart, and kidneys were carefully dissected out and weighed.

### Immunoblotting

Following euthanasia by CO_2_ inhalation, the mice were decapitated. The olfactory bulbs were quickly dissected out and snap frozen in liquid nitrogen; the tissues were stored at −80°C temporarily and then processed for protein extraction. The olfactory bulbs were homogenized in protein extraction buffer prepared in ultrapure water (50mM HEPES, 10% v/v glycerol, 1mM EDTA, 10mM NaF, 1mM activated Na_3_VO_4_, 150mM NaCl, 1% v/v Triton X 100, 0.1% w/v SDS), supplemented with cOmplete Mini EDTA-free Protease Inhibitor Cocktail tablets (cat # 11836170001; Sigma-Aldrich, Inc. St. Louis, MO) and PhosSTOP mini tablets (cat #4906837001; Sigma-Aldrich). Protein concentrations were determined through Bradford assay using Pierce BCA Protein Assay Kit (cat # 23227; ThermoFisher Scientific, Waltham, MA), measuring absorbance at 540nm. Samples (20 mg) were loaded on Invitrogen Novex 12% Tris-Glycine Plus Midi Gels (cat # WXP01226BOX; ThermoFisher Scientific) and ran for 50 minutes at 150 volts, and were then transferred onto nitrocellulose membranes at 4°C (25 volts for 60 minutes). The membranes were stained with Ponceau S to confirm equal loading. The membranes were blocked with 5% nonfat dried milk in 1X TBST buffer and then incubated overnight with one of the following antibodies, prepared in 5% nonfat dried milk, at 4°C: (1) Insulin/IGF-1 Signaling Pathway Antibody Kit (cat# 42022; Cell Signaling Technology, Danvers, MA), (2) human α-synuclein (cat # ab138501; abcam, Waltham, MA), (3) PSD-95 (cat # ab18258; abcam), (4) Synaptophysin (cat # S5768; Sigma-Aldrich), or (5) β-actin (JLA20; cat # AB_528068; DSHB, University of Iowa). The bands were visualized using Pierce ECL Western Blotting Substrate (Cat # 32209; ThermoFisher Scientific). Optical density measurements of the bands were performed using NIH ImageJ software, and the optical density values were normalized to that of β-actin.

### Statistics

Statistical analyses of the data were performed in R version 3+ (Vienna, Austria) or GraphPad Prism 10 (GraphPad Software, Inc.). As specified in the legend for each figure, the following statistical tests were used: independent unpaired t-tests, two-way and three-way repeated measures ANOVA, mixed effects model, and Log-rank (Mantel-Cox). All sets of continuous data were tested for normality using the Shapiro-Wilk test, and fewer than 5% of the tests concluded that the set was non-normal at the 0.05 significance level, confirming that the data sets met the assumption of a normal distribution. Results were expressed as mean ± SEM, and differences in mean were considered significant at *P* < 0.05.

## Data Availability

All raw data and any additional information on methodology or data analyses used in the current study will be available upon request to the corresponding authors.
